# Honey-inspired antimicrobial hydrogels resist bacterial colonization through twin synergistic mechanisms

**DOI:** 10.1038/s41598-020-72478-6

**Published:** 2020-09-25

**Authors:** Tiffany Zhang, Yue Qu, Pathiraja A. Gunatillake, Peter Cass, Katherine E. S. Locock, Lewis D. Blackman

**Affiliations:** 1 CSIRO Manufacturing, Research Way, Clayton, VIC 3168 Australia; 2 Chimie ParisTech, Rue Pierre et Marie Curie, 75005 Paris, France; 3 Infection and Immunity Program, Department of Microbiology, Monash Biomedicine Discovery Institute, Monash University, Clayton, VIC 3800 Australia; 4 Department of Infectious Diseases, The Alfred Hospital and Central Clinical School, Monash University, Melbourne, VIC 3004 Australia

**Keywords:** Biomaterials, Soft materials, Biocatalysis, Antimicrobials

## Abstract

Inspired by the interesting natural antimicrobial properties of honey, biohybrid composite materials containing a low-fouling polymer hydrogel network and an encapsulated antimicrobial peroxide-producing enzyme have been developed. These synergistically combine both passive and active mechanisms for reducing microbial bacterial colonization. The mechanical properties of these materials were assessed using compressive mechanical analysis, which revealed these hydrogels possessed tunable mechanical properties with Young’s moduli ranging from 5 to 500 kPa. The long-term enzymatic activities of these materials were also assessed over a 1-month period using colorimetric assays. Finally, the passive low-fouling properties and active antimicrobial activity against a leading opportunistic pathogen, *Staphylococcus epidermidis*, were confirmed using bacterial cell counting and bacterial adhesion assays. This study resulted in non-adhesive substrate-permeable antimicrobial materials, which could reduce the viability of planktonic bacteria by greater than 7 logs. It is envisaged these new biohybrid materials will be important for reducing bacterial adherence in a range of industrial applications.

## Introduction

Biofouling puts significant financial strain on a wide range of industrial processes and applications, including marine fouling on the hulls of ships and in pipes used for food processing or waste water treatment, and in healthcare applications^1–3^. Typical fouling agents range from proteins, through to prokaryotes (bacteria) and eukaryotic organisms such as algae and fungi, to larger marine life such as barnacles and mussels^2,3^. Fouling is a multi-step process proceeding through the formation of a protein layer, physically adsorbed to the surface within a few seconds of exposure to the media. In the case of microbial fouling, this proteinous conditioning layer allows attachment of single organisms to the surface, which induces phenotypical changes to allow for cell division and the development of microcolonies, as well as the production of extracellular polymeric substances (EPS)^4^. The accumulation of these microcolonies then allows for the formation of a mature biofilm, which can either contain a single species, or more commonly contain numerous components from multiple species. These microorganisms can then detach from the surface and, after dispersal into the surrounding environment, colonize newly conditioned surfaces^1,2,5^. Biofilms account for 65% of all bacterial infections^6^ and can readily form on medical devices resulting in the rise of healthcare-associated infections^5,7^.

A vast wealth of literature exists aimed at tackling the initial attachment of proteins and microbial fouling agents through the use of low-fouling surfaces^1–3,5,8,9^. These include hydrophilic polymer coatings and hydrogels, such as those based on poly(ethylene glycol) (PEG) and zwitterionic polymers among others^9–19^, as well as superhydrophobic surface coatings^3,20^, and those based on low-fouling surface topographies, such as those that mimic shark skin^21,22^ or other natural low-fouling patterns^23^. Furthermore, others have attempted to combine these materials with components that have active antimicrobial properties, such as to prepare materials with two synergistic mechanisms; fouling resistance plus antimicrobial properties^5,24^. Outside of dual polymer or polymer–metal composite materials, attention has also turned towards using enzymes as an active protein-degrading or antimicrobial component^25–27^. For example, Chiao and co-workers developed PEG-based hydrogel coatings with immobilized protease, which showed resistance towards fouling by model proteins using two synergistic mechanisms; both the intrinsic low-fouling properties of the PEG material and enzymatic protein degradation^28^. Similarly, lysostaphin, an enzyme which can break down the pentaglycine crosslinks in staphylococcal peptidoglycan, has been incorporated into injectable PEG-based hydrogel materials for the treatment of bacterial orthopaedic infections^29^.

Looking closer at nature, honey exhibits natural preservative properties and has been used in a variety of healthcare-associated applications including wound healing and the treatment of skin conditions^30–33^. One active antimicrobial component of honey is glucose oxidase (GOx)^34^, a 160 kDa homodimer enzyme, which facilitates the conversion of glucose into 1,5-gluconolactone, whilst producing antimicrobial hydrogen peroxide as a by-product^35^. GOx is also listed as being “Generally Regarded as Safe” by the Food and Drug Administration. Peroxide-producing enzymes such as GOx and cellobiose dehydrogenase (CDH)^27,36^ show an advantage over other antimicrobial enzymes^25^ or peptides^37,38^, which typically degrade or disrupt bacterial membranes or EPS, in that they do not require direct contact with bacteria in order to exhibit antimicrobial activity. For example, our group recently reported the preparation of semi-permeable nanoparticles with encapsulated GOx, which retained broad-spectrum antimicrobial activity in solution against a range of Gram-positive and Gram-negative bacteria in response to glucose^39^. Therefore, materials can be designed to combine peroxide-producing enzymes with low-fouling materials to mask the enzyme from the bacteria. Without protein exposed on the material surface, a reduced prevalence of biofilm formation can be achieved without significantly compromising the enzyme’s antimicrobial activity. For example, Ulbricht and co-workers developed zwitterionic layer-by-layer coatings, which entrapped CDH through electrostatic interactions to produce low-fouling surfaces. When these were deposited onto catheter materials, they led to the production of antimicrobial hydrogen peroxide, and a subsequent reduction in bacterial colonization^27^. Meyer and co-workers developed promising xylene-based coatings containing GOx, which could produce hydrogen peroxide in sea water and resulted in significantly reduced barnacle and tunicate coverage on rafts coated with this material in a North Sea field trial^40^. Very recently, Zanuy, Shalev and Reches et al*.* developed self-assembled tripeptide particles, which reduced *Escherichia coli* colonization when adhered to a surface. These were co-assembled with GOx to prepare surfaces with both antimicrobial and low-fouling properties^26^. Whilst these materials were highly effective in the short term, no discussion regarding the long-term stability of the coating was included in this study. Furthermore, the somewhat challenging synthesis and lack of proteolytic stability of tripeptides could limit the versatility of this approach in certain applications. GOx has also been extensively incorporated into hydrogel materials for a wide range of other applications, including glucose sensors^41–44^, glucose-swellable materials^45,46^, redox hydrogel-modified electrodes^47,48^, and drug delivery depots^49,50^.

**Figure 1:**
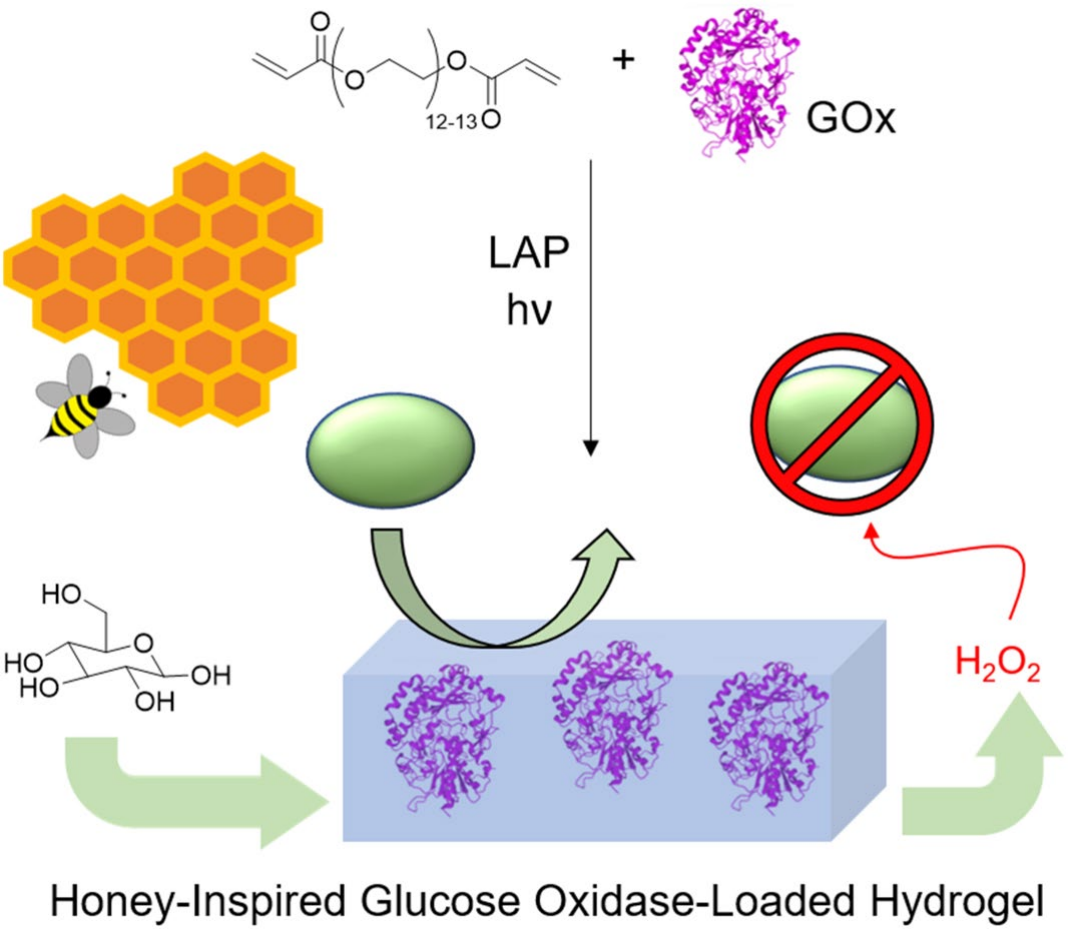
Preparation of the biohybrid GOx-PEGDA hydrogels. A scheme is depicted showing the synergy of combining low-fouling materials with non-contact killing antimicrobial enzymes. Protein structures were obtained from RSCB Protein Data Bank^51^ and visualized using the Mol* software^52^. Honeybee graphic adapted with permission from Blackman et al^39^. Copyright 2020 American Chemical Society.

Inspired by the antimicrobial properties of honey, herein we have prepared low-cost biohybrid composite hydrogel materials, which combine the synergistic benefits of low-fouling biocompatible polymer components with the antimicrobial properties of an encapsulated antimicrobial enzyme, GOx (Fig. 1). The mechanical properties and longevity of these materials over a one-month period was investigated, along with their interaction with a common biofouling human pathogen, *S. epidermidis*. These promising materials show exciting potential to reduce fouling in a range of industrial and biomedical applications.

## Results and discussion

Both empty hydrogel materials, and those loaded with GOx, were prepared using a photoinitiated curing methodology using a 700 g mol^−1^ poly(ethylene glycol) diacrylate monomer and LAP as a photoinitiator. A range of solids contents were investigated, from 5 to 20 wt%. It was hypothesized that increasing the network density would increase the stiffness of the hydrogel materials but also would limit the influx and efflux of enzymatic products and substrates, as well as the diffusive leakage of the protein from the material. Therefore, this range was investigated to find an optimal trade-off, giving rise to materials with high enzymatic activity but low protein leakage, as well as having acceptable mechanical properties.

### Longevity of the enzymatic activity

**Figure 2:**
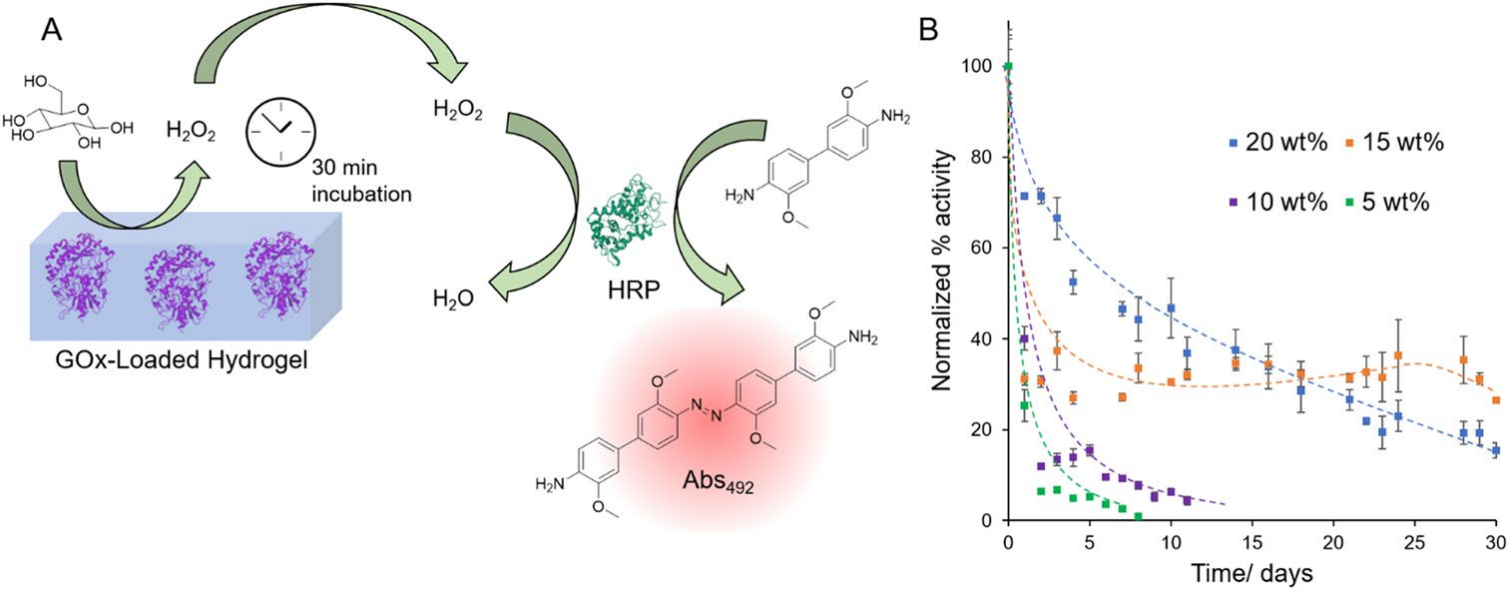
(**A**) Schematic of the activity assay whereby the hydrogels were incubated in a glucose solution for 30 min to allow for the production of H_2_O_2_. The [H_2_O_2_] in an aliquot of this solution was then quantified using a colorimetric assay utilizing horseradish peroxidase (HRP) and 3,3′-dimethoxybenzidine. Protein structures were obtained from RSCB Protein Data Bank^51,53^ and visualized using the Mol* software^52^. (**B**) Activities of the gels with time, each normalized to their day 0 activity. Dotted lines are shown to guide the eye. The error bars show standard deviation from 3 replicates.

One limitation of honey itself as a coating material is its poor longevity in aqueous environments. An important aspect for practical applications is the material’s ability to produce and release the antimicrobial small molecule peroxide species from glucose that had diffused into the gel, but still retain the active macromolecular enzyme GOx inside the gel network for an extended period of time. After their preparation, the enzyme-loaded hydrogels were soaked in PBS for 2 h to remove loosely bound GOx from the gel surface. The activity of the enzyme-loaded hydrogels was then assessed after soaking in PBS for up to one month with daily solvent changes. At each solvent change, the gels were washed with fresh PBS and a colorimetric assay used to determine the concentration of hydrogen peroxide released after a 30 min incubation period with a glucose solution (Fig. 2A). The activity was then normalized relative to the activity measured after the initial 2 h soaking period on day 0 of the experiment. Non-normalized data showing the absolute concentration of produced hydrogen peroxide is shown in the Supporting Information (Figure S2). It can be seen in Fig. 2B and Figure S2 that whilst the weaker 5 wt% and 10 wt% gels showed the highest initial activity on day 0, this activity rapidly decreased owing to leakage of GOx from the hydrogel. Conversely, whilst the 15 wt% and 20 wt% showed only moderate initial activities, this was retained for a much longer period. This could be explained by the loosely crosslinked hydrogels having larger effective pore sizes than those with a tighter network, thereby allowing faster diffusion of the macromolecule from the gel. Therefore, the 15 wt% and 20 wt% gels showed optimal properties in retaining the encapsulated protein whilst still allowing for acceptable substrate and product diffusion from the gel. The retention of activity past a 30-day period highlights the potential of these materials for low-fouling industrial applications.

### Characterization of mechanical properties

**Figure 3:**
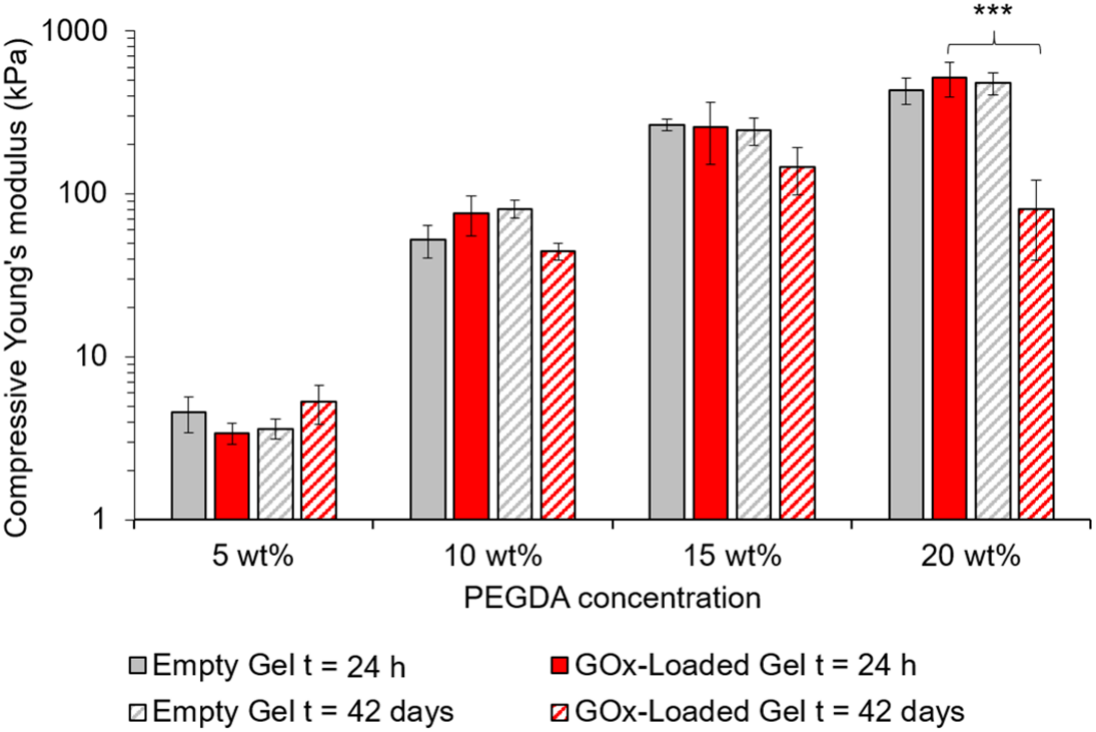
Mechanical properties of the empty and GOx-loaded hydrogels after soaking in PBS for 24 h and 42 days. An unpaired t-test (GraphPad) determined a *p* value of 0.0025 showing statistical significance between the 20 wt% GOx-loaded hydrogel after 24 h and 42 days, as indicated by asterisks.

The mechanical properties of the swollen hydrogels were investigated using an unconfined compressive mechanical analysis test. The compressive Young’s modulus was determined by monitoring the compressive stress up to 7% strain. It can be seen in Fig. 3 that, as expected, increasing the density of the hydrogel network increased the compressive Young’s modulus of these materials, which we attribute to the formation of greater chemical cross-linked junctions with increasing amounts of difunctional monomer. It is of note that in these freshly-prepared hydrogels, the incorporation of the antimicrobial enzyme does not significantly modify the mechanical properties of these materials.

To investigate how robust these materials were to the leakage of the enzyme from the gels, the hydrogels were also assessed after a 42-day soaking period. For the 5, 10 and 15 wt% PEGDA gels, the Young’s modulus remained constant after this period of soaking, however for the 20 wt% gel, an order of magnitude decrease in the Young’s modulus was observed in the case of the GOx-loaded gels (Fig. 3). Whilst the deteriorating mechanical properties of the 20 wt% gel over this time period should therefore be considered when utilizing stiff hydrogels with leachable macromolecular components, the mechanical properties of all the hydrogels investigated were nevertheless found to be sufficient for handling for up to a six-week period.

### Synergistic antimicrobial and low-fouling properties of GOx-loaded hydrogels

**Figure 4:**
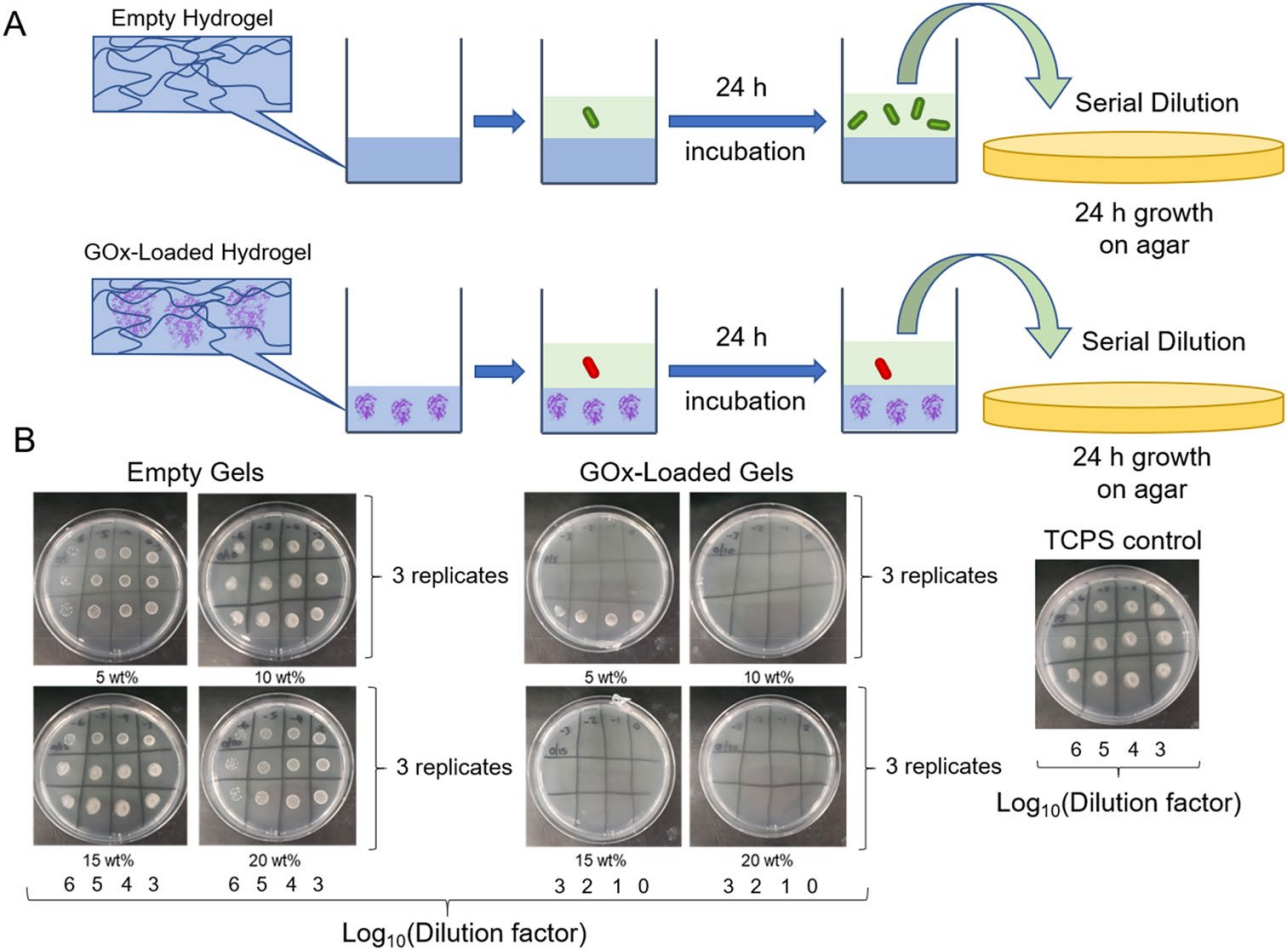
In vitro antimicrobial assessment of the hydrogels. (**A**) Schematic of the antimicrobial assay protocol. Protein structures were obtained from RSCB Protein Data Bank^51^ and visualized using the Mol* software^52^. Generic shapes adapted with permission from Blackman et al^39^. Copyright 2020 American Chemical Society. (**B**) Nutrient agar plates with 20 µL spots of different dilutions of bacterial cultures exposed to the empty and GOx-loaded gels. For each plate, horizontal rows indicate the 3 biological replicates and vertical columns show the serial logarithmic dilutions. A bacterial culture exposed only to tissue culture poly(styrene) (TCPS) is also shown. Note that the spots for the GOx-loaded gels are 1000-fold more concentrated than the empty gels and control.

As discussed, the combination of two distinct mechanisms; the active antimicrobial activity gained from the production of hydrogen peroxide and the low-fouling properties imparted from the swollen hydrophilic polymer component of the gel, is important for reducing bacterial colonization. To assess both of these properties, the interaction of these gels with *S. epidermidis*, a common gram-positive bacterial pathogen well-known for its ability to readily form robust biofilms on a range of surfaces^54^, was investigated. Both empty and GOx-loaded hydrogels were assessed for their active antimicrobial properties against this strain. The gels were first incubated in PBS for a 5-day period because this was deemed to be a time when the activity of each of the gels reached a relatively stable level (Fig. 2B). After washing and sterilization, the gels were then incubated with media pre-inoculated with *S. epidermidis*. After a 24 h growth period, viable counts were carried out to determine the antimicrobial activity of hydrogel against planktonic cells (Fig. 4A). It can be seen in Fig. 4B that full growth of *S. epidermidis* was observed for the empty gels, indicating that the gel material itself showed no active killing properties. However, cultures exposed to each of the GOx-loaded gels showed no viable colonies at any dilution, suggesting a > 7-log reduction in bacterial counts relative to the 20 wt% hydrogel formulation without GOx (quantified data is shown in Figure S3). This exciting result shows that such biohybrid composite materials are able to actively kill planktonic bacteria and hence show promise in a wealth of antimicrobial applications. Note that whilst no additional glucose was added, the endogenous glucose media concentration present was measured to be 0.344 mg mL^−1^, or 1.9 mM, using a calibrated glucose sensor (see experimental). This indicates that the hydrogels were able to rapidly convert the available glucose from the media into the required level of hydrogen peroxide for antimicrobial activity, as expected from the colorimetric assay data. Note that the glucose content used in the antimicrobial assay (1.9 mM) is well below that of blood (up to 7.8 mM)^55^, or food beverage wastewater (typically 330–660 mM depending on beverage)^56^. The minimum inhibitory concentration (MIC) of H_2_O_2_ itself was determined to be 0.5 mM against *S. epidermidis*. It can be seen in Figure S4 that when using the 15 wt% hydrogels, near complete consumption of glucose was observed after 18 h across a range of glucose concentrations between 0.3 and 1.9 mM. Therefore, we anticipate that the minimum glucose concentration required for antimicrobial activity is close to, or slightly higher than, the MIC of hydrogen peroxide (0.5 mM). However, it should be noted that the biocidal activity of produced H_2_O_2_ is likely to play a synergistic role with the bacteriostatic mechanism of glucose consumption (i.e. starvation) when using GOx, owing to GOx’s ability to deplete available glucose nutrient sources, which are required for bacterial growth.

**Figure 5:**
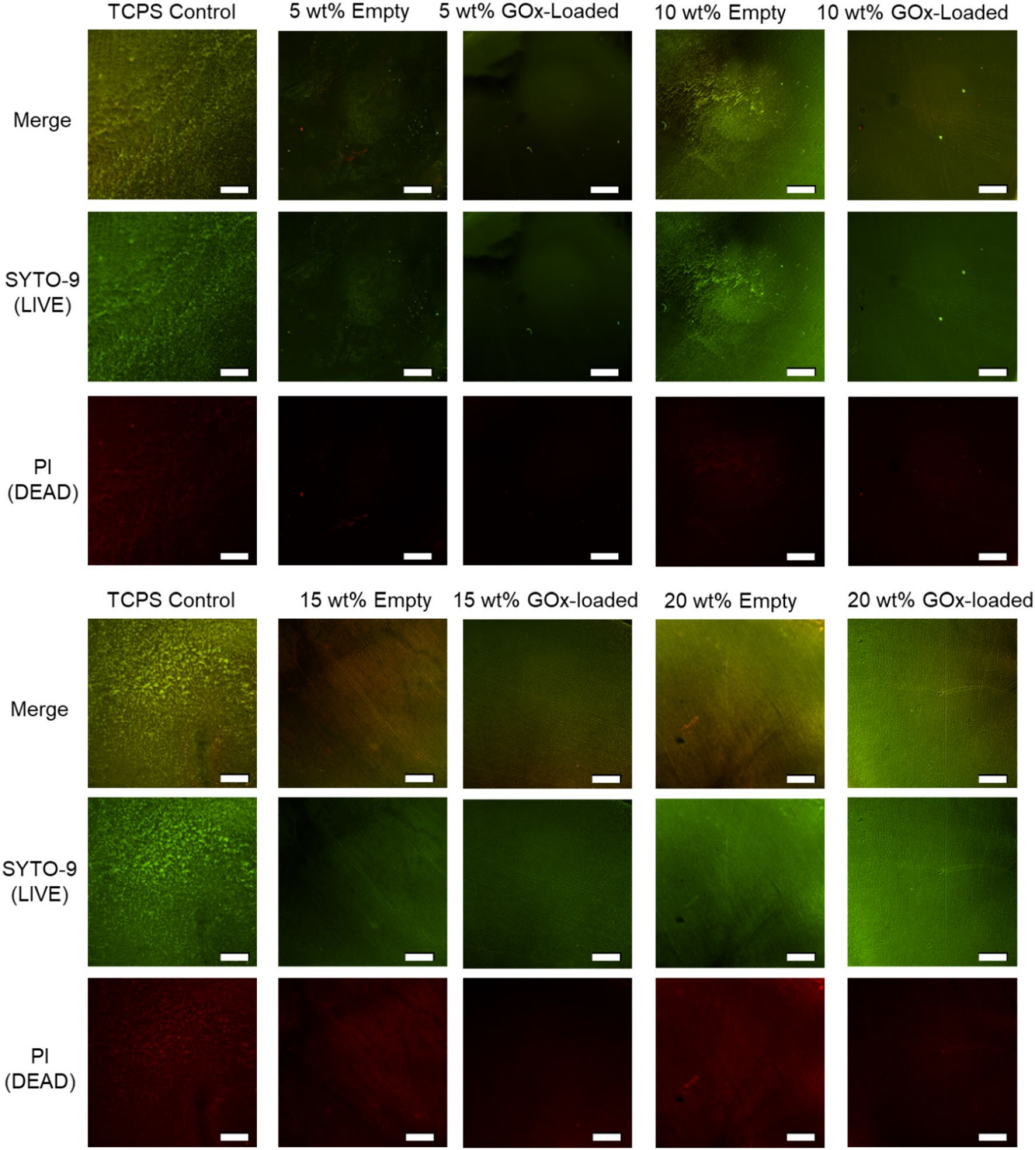
Representative fluorescence microscopy images of the empty and GOx-loaded hydrogels after 24 h incubation with *S. epidermidis* after staining with a *Baclight* LIVE/DEAD stain. Two regions of a control surface of tissue culture poly(styrene) (TCPS) are also shown for comparison. The scale bar represents 500 µm.

Aside from being endowed with active killing properties towards planktonic bacteria, one other important aspect for a number of applications is the material’s resistance to the formation of microbial biofilms. Therefore, a bacterial bioadhesion assay was performed on both empty and GOx-loaded hydrogels. Since it was established that the GOx-loaded hydrogels could actively kill bacteria and inhibit proliferation, it was important to also demonstrate that the hydrogel material itself had intrinsic low-fouling properties in the absence of the enzyme. After incubating with *S. epidermidis* for 24 h, the bacterial cells adhered to the hydrogels were stained using a *BacLight* LIVE/DEAD stain, which also allowed for the determination of viability of adhered bacteria. A control surface of tissue culture poly(styrene) (TCPS), which was expected to show a high degree of bacterial colonization, was also included in this assay. As shown in Fig. 5, the empty hydrogels indeed showed excellent low-fouling properties relative to the tissue culture poly(styrene) control. Note that owing to the low attachment of bacteria to the hydrogel surface, the bactericidal activity against adherent bacteria was not able to be investigated. Taken together, these results indicated that whilst the encapsulated GOx enzyme facilitates active killing properties towards planktonic bacteria, the PEGDA hydrogel material itself also imparts low-fouling properties, affording dual synergistic mechanisms for the reduction of bacterial colonization in the biohybrid composite hydrogels. The low-fouling properties of these materials is also in agreement with other literature reported for poly(ethylene glycol)-based hydrogels^18^. As expected, the hydrogels loaded with GOx also showed low-fouling properties, highlighting the ability for the gel to shield the protein from the media, thus reducing the prevalence of a conditioning layer for bacterial attachment.

## Conclusions

New honey-inspired materials for reducing fouling in industrial applications have been designed, which combine the synergistic mechanisms of low-fouling properties coupled with an active antimicrobial-producing enzyme. The longevity of the antimicrobial-producing activity and the mechanical properties of these materials were investigated using a range of complementary techniques. Specifically, PEGDA hydrogels prepared at 15 and 20 wt% were identified as having optimal mechanical properties as well as improved retention of the antimicrobial enzyme, whilst still allowing diffusion of the small molecule substrates and products necessary to enact the gel’s antimicrobial activity. Each gel in the series was shown to be low-fouling against a highly efficient biofouling bacterium, *S. epidermidis*, including those absent of the active antimicrobial component, whilst the GOx-loaded gels caused a > 7-log reduction in viable planktonic bacteria. This highlights the synergy of having both low-fouling and active antimicrobial components in these biohybrid composite materials. It is envisaged that these materials will be important in reducing biofouling in numerous industrial applications, including in food technology and wastewater management, as well as for biomedical and marine applications.

## Methods

### Materials

Poly(ethylene glycol) diacrylate (PEDGA, *M*_n_ = 700 g mol^−1^), and 3,3′-dimethoxybenzidine (DMB) were purchased from Sigma Aldrich and used as received. Glucose oxidase (type VII) from *Aspergillus niger* (GOx) and horseradish peroxidase (type VI) were purchased from Sigma Aldrich, dissolved in phosphate buffered saline at 200 U mL^−1^ and stored at − 20 °C in 100 µL aliquots. Fresh samples were defrosted on the day of use. Lithium phenyl-2,4,6-trimethylbenzoylphosphinate (LAP) was purchased from Tokyo Chemical Industry Co. and used as received. Dimethyl sulfoxide (DMSO) was purchased from Merck KGaA and used as received. Teflon hydrogel casters were purchased from Q-Gel, Switzerland.

Microbiological media Mueller–Hinton broth (MHB) and nutrient agar were purchased from Thermo Fisher Scientific (Oxoid) and prepared and sterilized according to the manufacturer’s instructions. *Bac*Light Live/Dead Viability kit containing propidium iodide (PI) and SYTO9 was purchased from Invitrogen and used according to the manufacturer’s instructions. *S. epidermidis* ATCC 35984 (RP62A) was used for all microbiological assays.

### Preparation of empty and GOx-loaded hydrogels

PEGDA (5, 10, 15 or 20 wt%) and LAP (0.05 wt%) were dissolved in pH 7.4 PBS. For GOx-loaded gels, GOx (20 U mL^−1^) was also included in this solution. 100 µL of this solution was cured in a Teflon caster at a wavelength of 320–500 nm and 60 mW power for 15 min to give clear, solid hydrogel disks, each of roughly 1.4 mm thickness.

### Activity of the empty and GOx-loaded hydrogels

After soaking in PBS (700 μL) for 24 h, supernatants were removed from the hydrogels and the hydrogels were washed with fresh PBS (700 μL). The hydrogels were soaked for 30 min in a glucose solution (0.6 mM, 1 mL) at 37 °C with agitation at 75 rpm. The solutions exposed to the gels (140 μL) were transferred to a 96-well plate and horseradish peroxidase (HRP) in PBS (2 U/mL, 20 μL) and 3,3′-dimethoxybenzidine (DMB) in 50% PBS/DMSO (2.5 mg/mL, 40 μL) were added and the absorbance at 492 nm was measured as a function of time. The absorbance of each of the samples was allowed to equilibrate for 5 min to provide stable absorbance values. These were then compared to a calibration performed under identical assay conditions but substituting the test solutions with known concentrations of H_2_O_2_. This assay was performed for up to 30 days.

For investigation of activity at various glucose concentrations, 15 wt% GOx-loaded hydrogels were prepared as above, and each was soaked in PBS (10 mL) for 24 h at 37 °C at 75 rpm shaking. After discarding the solution and rinsing with further PBS, the hydrogels were separately immersed in either 1.9 mM, 0.95 mM, 0.60 mM, or 0.30 mM glucose in PBS (1 mL per hydrogel) for 18 h at 37 °C at 75 rpm shaking. After this time, 100 μL of each exposed solution was diluted with 900 μL PBS to form the test solution. 140 μL test solution was transferred to a 96 well plate. HRP in PBS (2 U/mL, 20 μL) and DMB in 50% PBS/DMSO (2.5 mg/mL, 40 μL) were added and the absorbance at 492 nm was measured as a function of time. The absorbance of each of the samples was allowed to equilibrate for 5 min to provide stable absorbance values, which were compared to a calibration performed in parallel under identical assay conditions but substituting the test solutions with known concentrations of H_2_O_2_.

### Mechanical analysis

Mechanical analysis was performed using a modified literature procedure^57^. Compression analysis was performed on an Instron 5500R Physical Test Machine in compression mode using a 15 mm diameter compression probe and a 100 N load cell. Data was collected using the BlueHill3 software. All the experiments were conducted in a constant 50.5% relative humidity and constant 22.5 °C temperature room.

Prior to each experiment, the pellet of gel was removed from its PBS soaking solution and lightly dried with a Kimwipe. The pellet thickness and diameter were measured with a digital Kincrome 150 mm (6″) vernier calliper. The pellet was placed at the centre of the compression plate. The compression probe was lowered down to almost touch the sample before balancing the load and resetting the gauge length.

The compressive stress (σ) and strain (ε) were calculated from the normal force (*F*_N_), extension (Δ*l*), measured gel area (*A*) and thickness (*l*) according to Eqs. (1) and (2).

1 $$\sigma = \frac{{F_{{\text{N}}} }}{A}$$

2 $$\varepsilon = \frac{\Delta l}{{l_{0} }}$$

The gel pellet was compressed to a 30% strain at a 0.3 mm/min rate. A pre-load of 0.01 N was reached at 0.1 mm/min prior to the compressive test. This experiment was conducted on freshly prepared hydrogels after an overnight soaking period. The experiment was also repeated on hydrogels left to soak in PBS (10 mL) at 37 °C with shaking at 75 rpm for 6 weeks after curing. For each gel, a relatively linear relationship between stress and strain was observed between 0 and 7% strain and this was used for the calculation of the compressive Young’s modulus (*E*) according to Eq. (3). Representative stress vs strain plots are shown in Figure S1.

3 $$E = \frac{\sigma }{\varepsilon }$$

### Assessment of antimicrobial and anti-biofilm activities

The empty and GOx-loaded gels were prepared and soaked for 5 days in PBS (10 mL) to allow for initial burst release of the GOx and remove any unencapsulated GOx. After this time, hydrogel samples were washed and sterilized by transferring to a sterile 24-well plate and irradiating with UV light for at least 1 h in a biosafety cabinet prior to the bacterial assay. *S. epidermidis* RP62A was grown on nutrient agar plates and a single colony was used to inoculate 5 mL of Mueller–Hinton broth (MHB) and grown overnight at 37 °C with shaking at 75 rpm. This culture was diluted to 10^6^ colony-forming units per mL using MHB and 0.5 mL was introduced to each sterilized sample well in a 24-well plate for 24 h at 37 °C with shaking at 75 rpm.

For the antimicrobial assay targeting planktonic cells, media exposed to the gels were serially diluted with PBS down to 10^–6^ of the original culture density. An aliquot of 20 µL from each dilution was plated on a nutrient agar plate and grown for 24 h at 37 °C under static conditions. All measurements were performed in triplicate.

For the bioadhesion assay, the cultured media was removed from the 24-well plate and the remaining hydrogels were washed with PBS (1 mL). Viability staining was achieved using a *Bac*Light LIVE/DEAD stain (0.5 mL) prepared in 0.9% saline according to the manufacturer’s instructions, before washing any free dye from the samples with PBS (1 mL). The samples were mounted onto glass slides prior to analysis by fluorescence microscopy. The slides were imaged with a Nikon Eclipse T*i* fluorescence microscope running Nikon Elements v5.01 software. Images were obtained at 40× magnification, 1.8× analogue gain and exposure time of 400 ms, with filters adapted to Ex: 465–495 nm/ Em: 515–555 nm for SYTO-9 and Ex: 540–580 nm/ Em: 600–660 nm for propidium iodide.

The glucose concentration in MHB was measured using a John Morris Group YSI 2950 Biochemistry Analyzer calibrated with standards of known glucose concentration. For determination of the MIC of hydrogen peroxide, a sterile dilution series of hydrogen peroxide in PBS (100 μL per well in a 96-well plate) was introduced to a 10^6^ colony-forming units per mL culture of *S. epidermidis* in MHB (100 μL per well), prepared as above. After growing overnight at 37 °C and 75 rpm, the absorbance at 600 nm was read and the minimum concentration required for complete growth inhibition was determined to be 0.5 mM.

## Supplementary information

Supplementary file1
