# Supplementary material for: Honey-inspired antimicrobial hydrogels resist bacterial colonization through twin synergistic mechanisms
Source: Sci Rep. 2020 Sep 25;10:15796. doi: 10.1038/s41598-020-72478-6 (PMC7519120; doi:10.1038/s41598-020-72478-6)
Supplement: Supplementary file 1 — Supplementary file1 [file 41598_2020_72478_MOESM1_ESM.docx]

**Supporting Information for “Honey-Inspired Antimicrobial Hydrogels Resist Bacterial Colonization Through Twin Synergistic Mechanisms”**

*Tiffany Zhang,^1,2^ Yue Qu,^3,4^ Pathiraja A. Gunatillake,^1^ Peter Cass,^1^ Katherine E. S. Locock^1^* and Lewis D. Blackman^1^**

*^1^CSIRO Manufacturing, Research Way, Clayton, VIC 3168, Australia*

*^2^Chimie ParisTech, Rue Pierre et Marie Curie, Paris,* *75005 France*

*^3^**Infection and Immunity Program, Department of Microbiology, Monash Biomedicine Discovery Institute, Monash University, Clayton, VIC 3800, Australia*

*^4^**Department of Infectious Diseases, The Alfred Hospital and Central Clinical School, Monash University, Melbourne, VIC 3004, Australia*

**Email:* [*Katherine.Locock@csiro.au*](mailto:Katherine.Locock@csiro.au) *(K.E.S.L.)*

**Email:* [*Lewis.Blackman@csiro.au*](mailto:Lewis.Blackman@csiro.au) *(L.D.B.)*


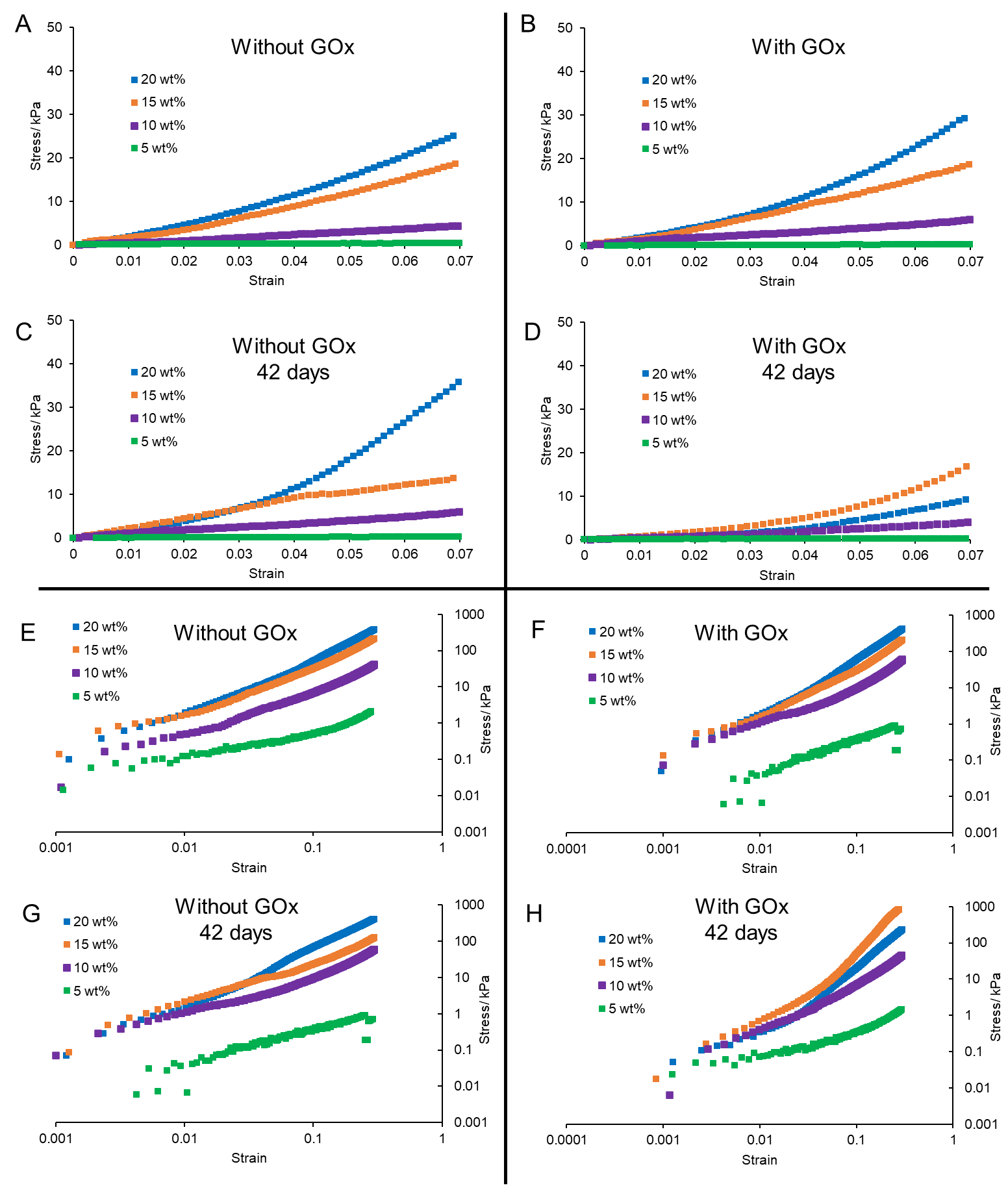


**Figure S1.** Representative compressive mechanical test data for empty PEGDA gels and GOx-loaded PEGDA gels as indicated. Linear plots showing up to 7% strain (A-D) and double logarithmic plots showing up to 30% strain (E-H) are shown. Data was obtained after soaking the gels in PBS at 37 °C for 24h (A-B and E-F) and 42 days (C-D and G-H). Moduli shown in Figure 2 were calculated from data collected up to 7% strain.


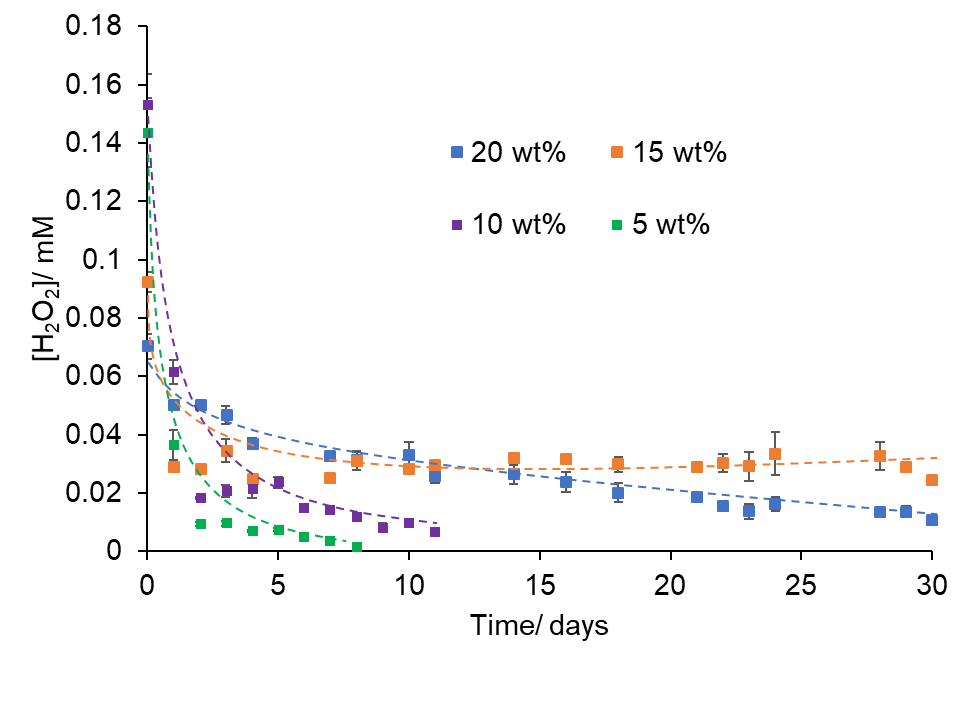


**Figure S2**. Non-normalized activity data for the GOx-loaded gels showing the change over time in absolute hydrogen peroxide concentration produced after 30 min incubation with 0.6 mM glucose. Dotted lines are shown to guide the eye.

**Figure S3.** Quantified log reduction in colony forming units for the GOx-loaded hydrogels against *S. epidermidis*, relative to growth observed in the presence of the 20 wt% empty hydrogel sample. Note that whilst three replicates were performed, data from one outlier replicate in the 5 wt% sample (as shown in Figure 4) has been omitted from this calculation.


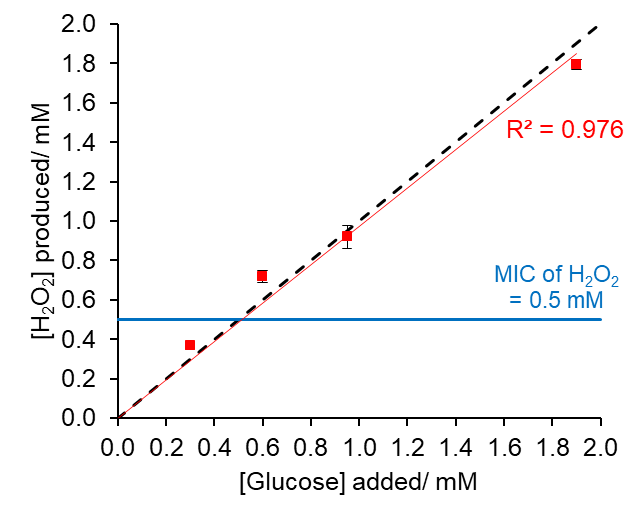


**Figure S4.** H_2_O_2_ production by the 15 wt% GOx-loaded hydrogels after 18 h at 37 °C and 75 rpm, as a function of glucose concentration. Error bars represent standard deviation across three replicate gels. A line of best fit is shown in red, as well as a black dashed line representing complete glucose consumption (i.e. [H_2_O_2_] = [Glucose]). The obtained minimum inhibitory concentration (MIC) for hydrogen peroxide against *S. epidermidis* is shown as a horizontal blue line. Hydrogels were pre-soaked in PBS for 24 h, followed by rinsing prior to the assay, to remove unencapsulated GOx.
